# The Nuclear Exosome Is Active and Important during Budding Yeast Meiosis

**DOI:** 10.1371/journal.pone.0107648

**Published:** 2014-09-11

**Authors:** Stephen Frenk, David Oxley, Jonathan Houseley

**Affiliations:** 1 Epigenetics Programme, The Babraham Institute, Cambridge, United Kingdom; 2 Proteomics Group, The Babraham Institute, Cambridge, United Kingdom; University College London, United Kingdom

## Abstract

Nuclear RNA degradation pathways are highly conserved across eukaryotes and play important roles in RNA quality control. Key substrates for exosomal degradation include aberrant functional RNAs and cryptic unstable transcripts (CUTs). It has recently been reported that the nuclear exosome is inactivated during meiosis in budding yeast through degradation of the subunit Rrp6, leading to the stabilisation of a subset of meiotic unannotated transcripts (MUTs) of unknown function. We have analysed the activity of the nuclear exosome during meiosis by deletion of *TRF4*, which encodes a key component of the exosome targeting complex TRAMP. We find that TRAMP mutants produce high levels of CUTs during meiosis that are undetectable in wild-type cells, showing that the nuclear exosome remains functional for CUT degradation, and we further report that the meiotic exosome complex contains Rrp6. Indeed Rrp6 over-expression is insufficient to suppress MUT transcripts, showing that the reduced amount of Rrp6 in meiotic cells does not directly cause MUT accumulation. Lack of TRAMP activity stabilises ∼1600 CUTs in meiotic cells, which occupy 40% of the binding capacity of the nuclear cap binding complex (CBC). CBC mutants display defects in the formation of meiotic double strand breaks (DSBs), and we see similar defects in TRAMP mutants, suggesting that a key function of the nuclear exosome is to prevent saturation of the CBC complex by CUTs. Together, our results show that the nuclear exosome remains active in meiosis and has an important role in facilitating meiotic recombination.

## Introduction

The exosome is a highly conserved exo- and endonuclease complex that degrades a wide variety of cellular RNAs. It is involved in the maturation of ribosomal RNA and some small RNAs [1]–[4], and is also responsible for 3′-5′ mRNA degradation in the cytoplasm [5], [6]. The exosome plays a major role in RNA quality control by degrading aberrant ribosomal RNAs, tRNAs, small RNAs and mRNAs, reducing the potential dominant negative effects of improperly processed RNA [1], [7]–[11]. The exosome exists in different forms in the nucleus and cytoplasm, with the nuclear exosome containing an additional exonuclease subunit, Rrp6. Rrp6 is required for 3′ end formation of some rRNA and snoRNA species, but unlike the core exosome is not essential [3], [12], [13]. Rrp6 orchestrates nuclear RNA surveillance functions of the exosome, mediating the transcription-site retention and degradation of mRNA that fails to be terminated, spliced, poly-adenylated or exported [10], [14]–[17].

Purified exosome has only weak nuclease activity *in vitro* and is targeted to substrates by various proteins and complexes including Rrp47, Mpp6 and the TRAMPs [9], [18]–[24]. The TRAMP complexes contain a poly(A) polymerase (Trf4 or Trf5), an RNA binding protein (Air1 or Air2) and a helicase (Mtr4) [9], [21], [22]; substrates are bound and polyadenylated by TRAMP most likely to help engage exosome activity, although the poly-adenylation activity of Trf4 is dispensable for degradation of many substrates [25]–[27]. Cryptic unstable transcripts (CUTs) form a large class of nuclear exosome substrates: these ephemeral RNAs are transcribed from the promoters of many protein coding RNAs, but are instantly degraded and remain almost undetectable in wild-type cells [9], [28]. CUTs are transcribed by RNA polymerase II and are co-transcriptionally bound by Nab3 and/or Nrd1 which recruit TRAMP and the exosome [29]–[31]. However, Nrd1 and Nab3 binding sites are not dramatically enriched in CUTs, leaving it somewhat unclear how they are selected for efficient degradation [18]. A few CUTs have ascribed functions, but it is unknown whether the majority of the ∼2000 CUTs encoded in the yeast genome have any function or whether degradation of these species is important [32]–[35]. Like the exosome, CUTs are highly conserved and equivalent species have been reported in human and plant cells [36], [37]. Loss of Trf4, the catalytic component of the TRAMP complex, causes dramatic CUT stabilisation but Trf4 is non-essential and *trf4*Δ mutants have mild phenotypes at normal growth temperatures [38]–[40].

It was recently reported that budding yeast degrade Rrp6 on entering meiosis, abrogating nuclear exosome function and stabilising a class of meiotic unstable transcripts (MUTs) [41]. Rrp6 degradation occurs across the first few hours of meiosis, coinciding with DNA replication and the induction of meiotic double strand breaks. This process may have a parallel in fission yeast where many meiotic genes are expressed during mitosis but are degraded by the exosome [42]. In mitosis, hexanucleotide motifs in meiosis-specific mRNAs are bound by the meiotic regulator Mmi1, which recruits a nuclear silencing complex that interfaces with the exosome [43]–[45]. Degradation of meiotic mRNA requires polyadenylation, and cells lacking the nuclear poly(A) binding protein Pab2 or carrying mutations in the canonical poly(A)-polymerase Pla1 accumulate meiotic transcripts during mitosis [46], [47]. Polyadenylation is required at multiple stages as meiotic mRNAs also accumulate in cells lacking Cid14, the fission yeast orthologue of Trf4, indicating that the TRAMP complex processes meiotic mRNAs for exosomal degradation [48]. Meiotic mRNA degradation must be efficiently suppressed on entry to meiosis; it is not clear precisely how this occurs but sequestration of Mmi1 into a complex of Mei2 and a ncRNA called meiRNA is strongly implicated [42], [49], [50].

Cells lacking Rrp6 almost never enter meiosis [41], suggesting that the nuclear exosome does have an important meiotic function despite the apparent degradation of Rrp6. However, this inability of *rrp6*Δ cells to enter meiosis prevents a direct assessment of meiotic exosome activity. Here we report the analysis of CUT degradation by the nuclear exosome in meiotic cells.

## Results

### CUT degradation in meiotic cells

**Figure 1:**
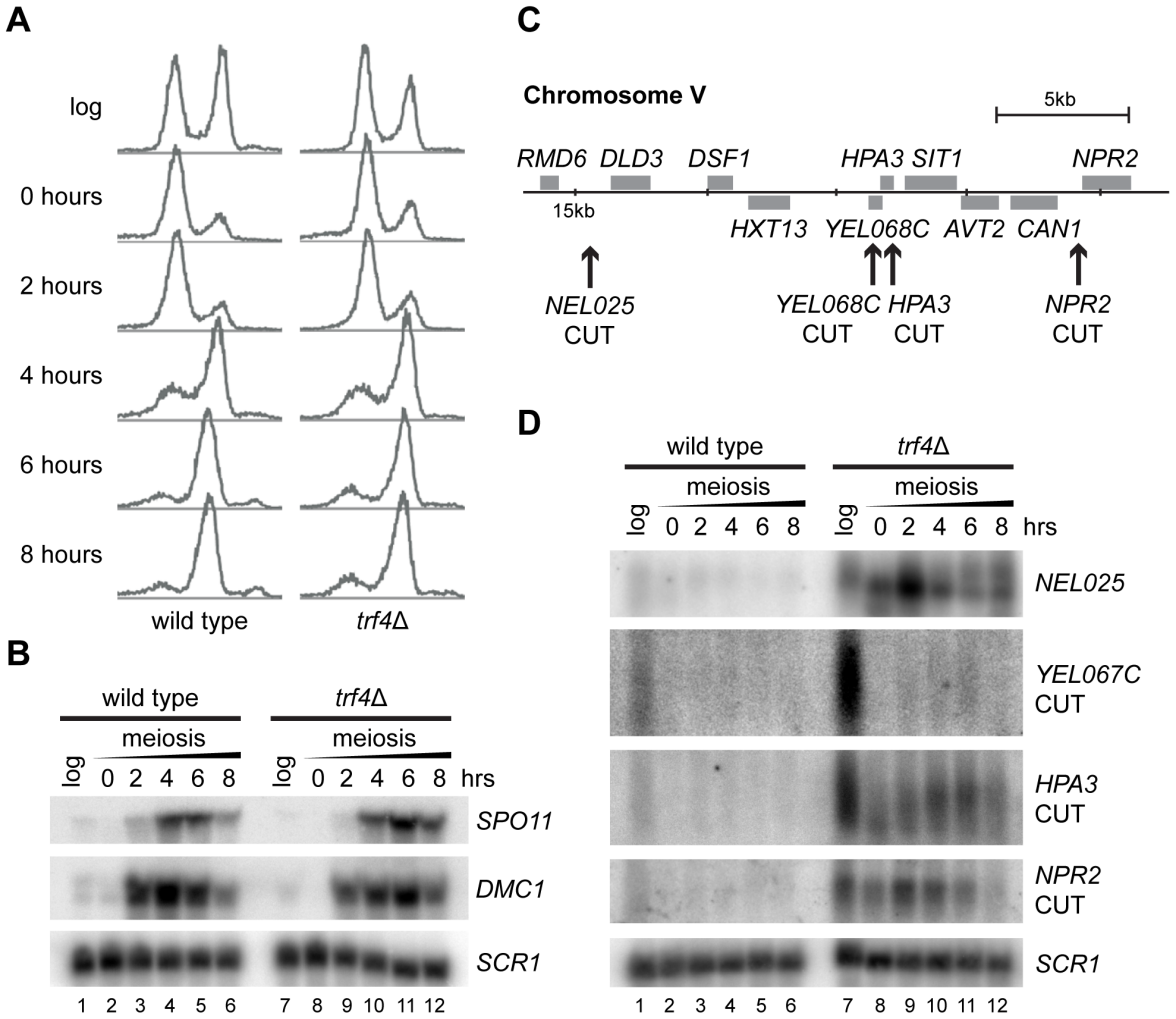
TRAMP mutants undergo normal meiosis despite CUT stabilisation. A: FACS analysis of DNA content in wild-type and *trf4*Δ mutants taken at 2 hour time-points during meiosis, showing that meiotic DNA replication is normal in *trf4*Δ cells. B: Northern analysis showing expression of meiosis-specific mRNAs *SPO11* and *DMC1* in wild-type and *trf4*Δ cells. Replicate northern blots of the same samples were prepared for each probe; a representative *SCR1* control is shown from the *SPO11* blot. Quantification for *SPO11* is given in Figure S1C. C: Schematic diagram of *NEL025* and adjacent CUTs at the *HPA3, YEL067C* and *NPR2* loci. D: Northern blot analysis of *NEL025* and adjacent CUTs in log-phase cells growing on YPA and at 2 hour time-points during meiosis. *SCR1* is shown as a loading control. Samples were run on replicate blots and each probed for a different CUT to avoid signal from inefficient stripping, a representative loading control is shown.

Mitotic cells lacking the TRAMP component Trf4 fail to degrade CUTs, but Trf4 is not essential for meiosis [51], [52] and so CUT stabilisation in *trf4*Δ cells provides a measure of meiotic exosome activity. We deleted *TRF4* in SK1, an *S. cerevisiae* strain background that undergoes synchronised meiosis under nitrogen starvation. Homozygous SK1 *trf4*Δ mutants were cold sensitive at 18° as observed in other strain backgrounds, but showed only a minimal growth defect at 30° and formed tetrads at the semi-permissive temperature 25° (Figure S1A,B). FACS analysis demonstrated that meiotic DNA replication was normal in *trf4*Δ cells at 25° (Figure 1A) and induction of key meiotic genes *SPO11* and *DMC1* occurred with similar dynamics to wild-type (Figure 1B and Figure S1C). Therefore, the TRAMP complex is not essential for meiosis.

We analysed meiotic expression of the well-characterised CUT *NEL025* and three surrounding CUTs identified by Neil *et al*. [53] (Figure 1C), two of which are stabilised in *rrp6*Δ mutants [54]. All four CUTs were readily detectable in SK1 *trf4*Δ mutants during logarithmic growth but were only just visible in wild type as expected (Figure 1D compare lanes 1 and 7). In meiotic *trf4*Δ cells, three out of four CUTs including both Rrp6-dependent transcripts were detected at similar levels to log phase, however the signal in wild-type cells was if anything reduced (Figure 1D, compare lanes 2–6 to 8–12), indicating that CUTs are expressed but unstable in meiotic cells.

**Figure 2:**
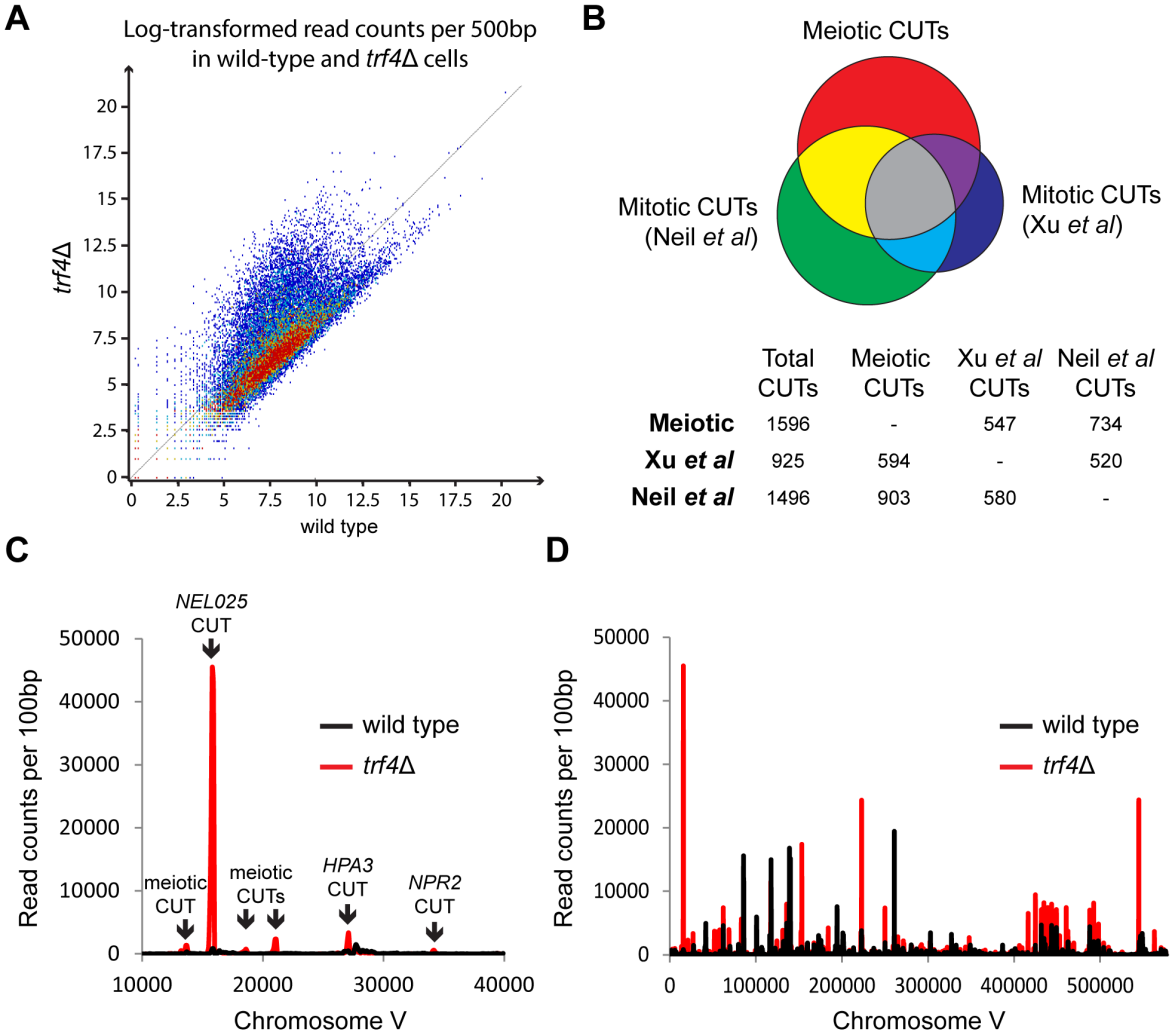
Genome-wide distribution of CUT transcripts during meiosis. A: Scatter plot of log-transformed read counts from Cbc2-associated RNA isolated from wild-type and *trf4*Δ cells after six hours of meiosis. Each point represents the read count from a single 500 bp genome region. B: Venn-diagram showing overlap between CUT transcripts identified in meiotic cells and published CUT datasets from mitotic cells [53], [54]. Actual numbers are given in the table below, note that overlapping counts are different depending on the direction of analysis as multiple CUTs defined in one dataset can map to a single locus in another dataset. For the Venn diagram, the number of overlapping CUTs was taken as the average of the values from both directions. C: Distribution of sequencing reads in 100 bp segments around the *NEL025* locus from meiotic Cbc2-associated RNA in wild-type and *trf4*Δ cells. This graph differs from Figure 2A in that actual read counts as opposed to log-transformed values are shown, the points in Figure 2A that correspond to this region are shown in Figure S2C. D: Distribution of sequencing reads across chromosome V, as in C.

*NEL025* and surrounding CUTs are not necessarily representative, and we therefore obtained meiotic transcriptomes of wild-type and *trf4*Δ cells for comparison with published mitotic CUT profiles. To increase the sensitivity of CUT detection we purified RNA associated with Cbc2, a component of the Cap Binding Complex (CBC) [55], [56] using the method of Neil *et al*. [53]. 2-step purification of Cbc2-TAP from meiotic cells was efficient (Figure S2A) and strongly enriched for *NEL025* RNA compared to 18S (Figure S2B). As expected, read counts for RNAseq libraries of Cbc2 immunoprecipitates from wild-type and *trf4*Δ cells after six hours of meiosis were skewed to higher numbers in *trf4*Δ than wild type, consistent with stabilisation of a broad range of CUTs throughout the genome (Figure 2A).

No exact CUT specification has been proposed so we annotated meiotic CUTs as transcripts >200nt that are over-represented >4-fold in the *trf4*Δ RNAseq data. These are stringent criteria compared to previous analyses [53], [54]. We also applied a noise cut-off to remove dubious calls from regions with very low read counts. By this definition, we annotated 1596 CUTs under active degradation in meiotic cells - a surprisingly high number but similar to that reported by Neil *et al*. for mitotic cells [53]. As shown in Figure 2B, 60% of the mitotic CUTs reported by Neil *et al*. and 64% of the mitotic CUTs reported by Xu *et al*. overlapped with meiotic CUTs [53], [54]. For comparison, only 56% of the mitotic CUTs discovered by Xu *et al*. were also discovered by Neil *et al*. suggesting that differences between meiotic and mitotic datasets stem primarily from technical variation and do not represent a major change in CUT metabolism. Importantly, Xu *et al*. identified CUTs based on stabilisation in *rrp6*Δ cells, so the fact that over half these CUTs are also unstable in meiotic cells strongly suggests that Rrp6 is functional during meiosis. These comparisons were performed using only reads which map uniquely to the genome as the microarrays used by Xu *et al*. and Neil *et al*. excluded non-unique regions, and were also mapped to the S288C rather than the SK1 genome, however, re-performing the analysis with non-unique reads included and mapping against the SK1 genome identified only 18 additional CUTs. A list of the meiotic CUTs identified in this analysis is given in Table S1. Together, these data demonstrate that TRAMP-mediated CUT degradation remains active during meiosis.

Cbc2 binds to the cap of all nascent RNA polymerase II transcripts, and we therefore expected that CUTs and mRNAs would be similarly represented in the Cbc2-immunoprecipitated material. However, the analysis of the actual read density across the region of chromosome V shown in Figure 1C, which contains 10 ORFs, was dominated by a small number of mainly intergenic peaks (Figure 2C). In fact, all the peaks visible in Figure 2C are CUTs, three of which are also present in mitotic cells and three are only expressed in meiosis. We plotted similar distributions over a number of complete chromosomes (Figure 2D and Figure S3); at this resolution, some highly expressed features were detected in the wild-type distribution but the largest and most numerous peaks were CUTs in *trf4*Δ cells. The apparent disparity between the data in Figures 2A and 2C,D stems from the fact that the read counts in Figure 2A have been log-transformed whereas Figure 2C,D show untransformed read counts; a version of Figure 2A highlighting the region in Figure 2C is shown in Figure S2C. In fact 39% of Cbc2-associated sequencing reads were found to derive from annotated CUT sites in *trf4*Δ cells compared to just 3% in wild-type. This enrichment did not represent a sequencing artefact as northern blotting of Cbc2 immunoprecipitated material also revealed a massive enrichment of *NEL025* compared to *ACT1* (Figure S2B). This suggests that CUTs could saturate the CBC if not efficiently degraded.

### Exosome composition is little changed in meiosis

**Figure 3:**
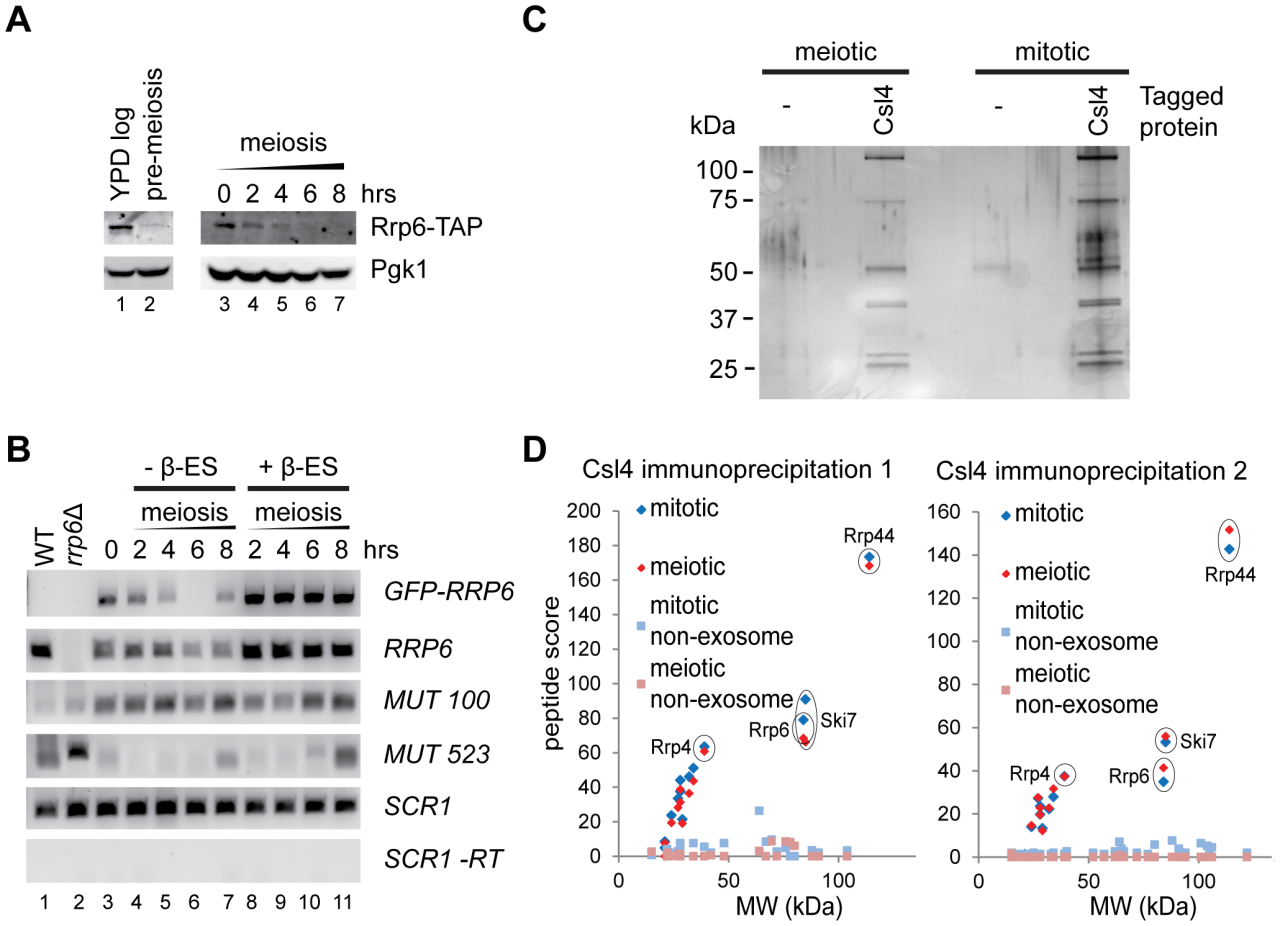
Exosome characterisation in meiotic cells. A: Western blot for Rrp6-TAP and Pgk1. Left-hand blot compares Rrp6 in log phase YPA and at the initiation of meiosis; right-hand blot, shown at different exposure, shows the gradual decline in Rrp6 levels across meiosis. B: Analysis of the effect of estradiol-induced GFP-Rrp6 over-expression on MUT stability. RT-PCR reactions for *RRP6* and MUTs were performed on RNA from cells without and with estradiol, compared to an *SCR1* loading control. *MUT 100* is expressed throughout meiosis whereas *MUT 523* is only expressed after 8 hours, neither is repressed by Rrp6 overexpression. C: Silver-stained protein gel showing Csl4-TAP immunoprecipitations from meiotic and mitotic cells, compared to purifications from untagged strains. Mitotic cells were grown on YPD, meiotic cells were harvested after six hours in SPO media. D: Plots of peptide score vs. molecular weight for proteins identified by mass spectrometry in two independent immunoprecipitation experiments. Non-yeast proteins and proteins also discovered in the untagged control sample were discarded, then the proteins were divided into exosomal and non-exosomal sets, both of which are displayed. Key exosome proteins including Rrp6 are highlighted.

To better understand the changes in Rrp6 activity during meiosis, we performed western blots for Rrp6-TAP in wild-type cells. As previously reported [41], Rrp6 levels decreased across meiosis (Figure 3A lanes 3–7), but this effect was minimal compared to the dramatic difference between log phase cells in YPD and pre-meiotic cells (dense cultures in YP acetate media) (Figure 3A lanes 1,2). Therefore, Rrp6 is already depleted at the start of meiosis so the effects of Rrp6 depletion on nuclear exosome activity should be detectable throughout meiosis.

Lardenois *et al*. suggested that Rrp6 protein levels are specifically reduced during meiosis through translational inhibition or proteolytic degradation [41]; we reasoned that if so then MUTs which become detectable early in meiosis should be destabilised by over-expression of Rrp6. To this end, we constructed an estradiol-inducible Rrp6 over-expression strain by introducing a heterozygous *GFP-RRP6* construct under a *GAL1* promoter into a strain expressing an ER-Gal4 fusion. This system allows estradiol-inducible expression of genes with a *GAL1* promoter during meiosis [57]. The *GAL1*-driven fusion was functional as dissected spores from this strain accumulated the characteristic 5.8S+30 rRNA fragment on glucose but not on galactose (Figure S4A). Meiosis was induced in this strain in the presence or absence of estradiol and expression of *RRP6* and two MUTs was analysed by RT-PCR. Although Rrp6 was very strongly induced and produced full length protein, the MUTs were not destabilised (Figures 3B, S4B), showing that reduced Rrp6 levels are not responsible for MUT stabilisation in meiosis.

To test for other changes in the meiotic exosome complex, we purified the exosome from mitotic and meiotic cells through a TAP tag on the core exosome subunit Csl4. Silver staining of the purified complexes revealed a similar banding pattern in meiotic and mitotic samples, although yields of the exosome were reproducibly lower from meiotic cells (Figure 3C). We performed the immunoprecipitation experiment using two different protocols and analysed the samples from both experiments by mass spectrometry. As expected, the complete core exosome was detected in all mitotic and meiotic samples but not in untagged controls, as were the exosome cofactors Rrp6 and Ski7 (Table 1). The one exception was Rrp43, which was not detected for reasons that remain unclear. Peptide scores obtained in these experiments are semi-quantitative and, to a first approximation, the peptide score of proteins at 1∶1 stoichiometry rises linearly with mass. Plots of peptide score versus mass show that this relationship holds true for core exosome subunits in all samples, whereas poorly associated non-exosomal proteins have very low peptide scores for their mass, indicating a very low abundance in the samples (Figure 3D). Ski7 and Rrp6 are associated with the cytoplasmic and nuclear exosome complexes respectively and have lower peptide scores than core subunits, as expected since both are sub-stoichiometric to the core exosome, but are still clearly separate from weakly associated non-exosomal proteins (Figure 3D, compare Rrp6 and Ski7 to core components Rrp44, Rrp4 and non-exosomal proteins). These plots provide no evidence that Rrp6 association with the core exosome changes dramatically between meiosis and mitosis, as the peptide score for Rrp6 is almost identical in both conditions (Figure 3D, highlighted Rrp6 signals). Mitotic exosome preparations contained various non-exosomal proteins, primarily involved in ribosome synthesis as expected given the importance of the exosome in this process. Only one meiosis-specific exosome associated protein was detected in both experiments: the RNA binding protein Rim4 which has recently been implicated in control of meiotic translation [58]; however we were unable to verify this interaction by co-immunoprecipitation despite repeated attempts using different tagged constructs. Therefore, although the meiotic exosome is less abundant than the mitotic exosome, it remains associated with Rrp6 and we did not detect any other differences that could explain MUT accumulation.

**Table 1:** Peptide scores for proteins isolated in exosome immunoprecipitations.

Identification	Function	Meiosis IP1	Meiosis IP2	Mitosis IP1	Mitosis IP2
Rrp44	exosome component	168	152	173	143
Ski7	exosome component	66	56	91	53
Rrp6	exosome component	68	41	79	35
Rrp4	exosome component	61	37	63	37
Rrp45	exosome component	44	32	51	28
Rrp41	exosome component	39	23	44	23
Csl4	exosome component	36	23	46	22
Mtr3	exosome component	31	19	37	20
Rrp40	exosome component	28	28	33	27
Rrp46	exosome component	19	15	24	14
Rrp42	exosome component	19	12	21	13
Lrp1	exosome component	8	0	9	1
Rim4	Meiosis RNA binding	6	0	0	0
Mpp6	exosome component	0	0	5	0
Pwp2	ribosome synthesis	0	0	3	3
Utp4	ribosome synthesis	0	0	3	3
Utp13	ribosome synthesis	0	0	2	2
Utp5	ribosome synthesis	0	0	3	3
Rps7A	ribosome component	0	0	1	1

Non-exosomal proteins were only included if detected in both immunoprecipitation experiments.

### TRAMP activity is required for normal meiotic DSB formation

**Figure 4:**
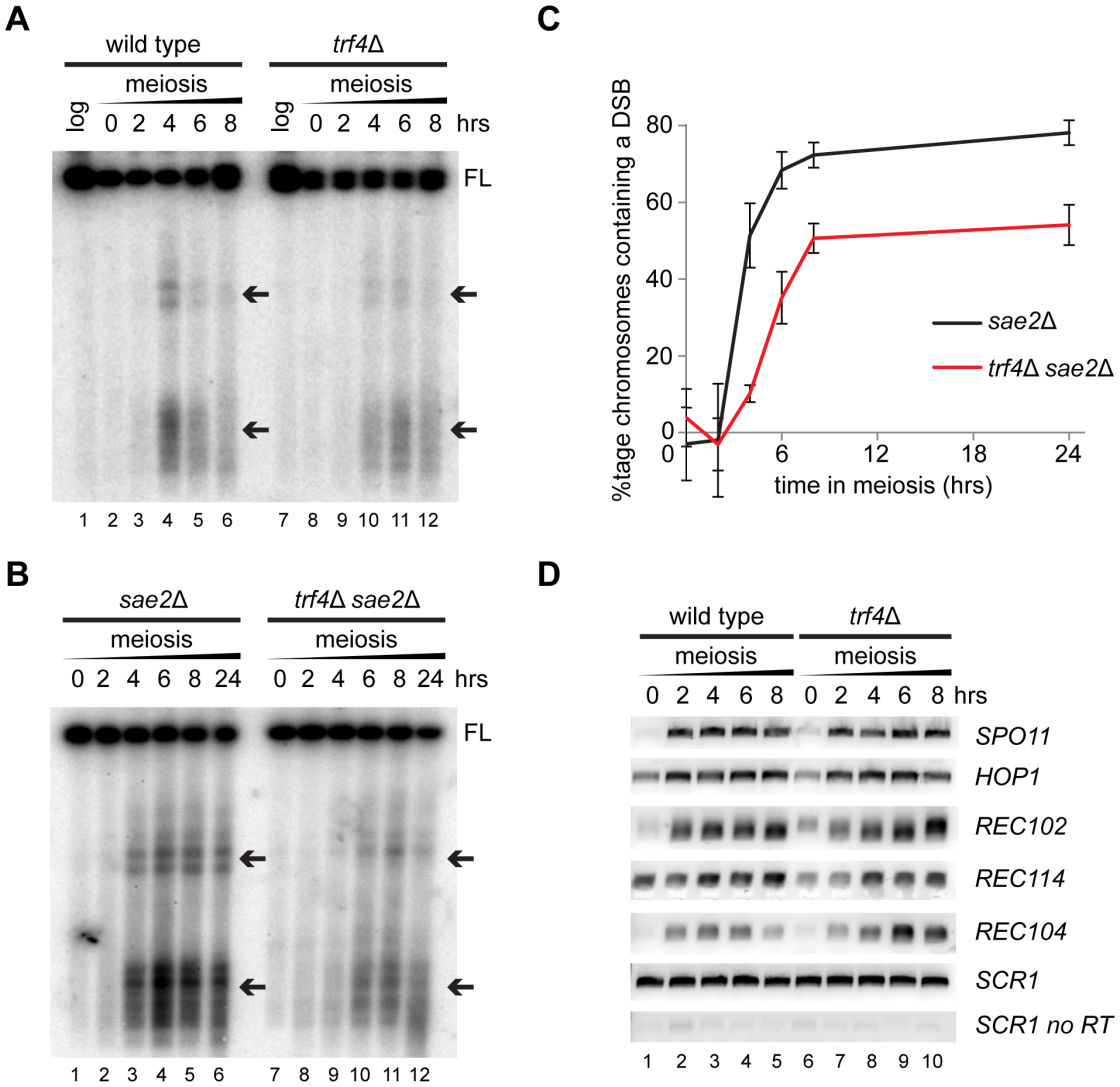
Meiotic DSB formation is defective in *trf4*Δ cells. A: PFGE analysis of transient DSBs in chromosome III during meiosis in wild-type and *trf4*Δ cells, truncated chromosome fragments caused by DSB formation are indicated by arrows. B: PFGE analysis showing the accumulation of cleaved chromosome III fragments during meiosis in *sae2*Δ and *sae2*Δ *trf4*Δ cells. C: Quantification of chromosome III DSBs in *sae2*Δ and *sae2*Δ *trf4*Δ cells. Error bars indicate ±1 s.e., * - p<0.05, *** - p<0.01 by student's *t*-test, n = 5. D: RT-PCR analysis of mRNA expression for selected meiotic recombination proteins in wild-type and *trf4*Δ cells.

Finally, we searched for any phenotype caused by lack of *TRF4* in meiotic cells; the TRAMP complex is highly conserved but clear phenotypes in TRAMP mutants tend to be weak leaving the importance of TRAMP function somewhat unclear. Since meiotic replication proceeded normally in *trf4*Δ cells, we analysed the next step: meiotic recombination. This process is initiated by the endonuclease Spo11, which cuts chromosomes at well-mapped hotspots. Transiently cleaved chromosome fragments are sufficiently abundant to be detected as truncated fragments migrating below the main chromosome band on PFGE gels probed for short chromosomes. PFGE analysis for cleaved fragments of chromosome III in wild-type cells revealed a transient peak after four hours of meiosis that was much weaker in *trf4*Δ mutants (Figure 4A, compare lanes 4 and 10). However, a transient peak in *trf4*Δ cells would be missed if it did not coincide with a sampling time, and we therefore repeated this assay in an *sae2*Δ background in which double strand breaks cannot be repaired [59], [60]. In *sae2*Δ cells, cleaved fragments accumulated between 2 and 8 hours of meiosis, reaching a plateau at 8–24 hours (Figure 4B lanes 1–6). This pattern was replicated in *sae2*Δ *trf4*Δ mutants but a significantly smaller percentage of chromosome III was cleaved (Figure 4B,C); this is important as chromosomes that do not form at least one DSB have a high likelihood of mis-segregation at meiosis I. Although Trf4 is an RNA processing protein, we could detect no defect in the expression of key recombination factors by RT-PCR (Figure 4D), suggesting that this was not caused by a specific gene expression defect. Therefore, although cells lacking TRAMP activity appear to progress normally through meiosis, they show significant defects in meiotic recombination.

## Discussion

Here we have reported a detailed analysis of TRAMP function during meiosis. We have demonstrated that TRAMP targets widespread CUTs for degradation in meiotic cells just as it does in mitotic cells. We have also shown that TRAMP facilitates meiotic DSB formation, providing an important physiological role for TRAMP activity.

It has been reported that meiotic cells undergo Rrp6 degradation, resulting in a loss of nuclear exosome function and the stabilisation of MUTs [41]. Such a process would be very surprising as the exosome is a highly conserved and ubiquitously expressed complex; Rrp6 is involved in ribosomal RNA synthesis and quality control [7], [13], [21], and many eukaryotes including *S. cerevisiae* perform ribosome re-synthesis during meiosis for which quality control mechanisms would seem vital [61]–[64]. We find that Rrp6 levels are much reduced in meiotic cells, but this appears to coincide with a general down-regulation of exosome levels in pre-meiotic cells grown to high density. Purified exosome from meiotic yeast contains Rrp6 and the meiotic exosome appears fully functional for CUT degradation. Furthermore, the re-expression of Rrp6 is insufficient to destabilise MUT transcripts, showing that the stabilisation of MUTs in meiosis cannot be attributed to a lack of Rrp6. In *S. pombe*, meiosis-specific mRNAs are degraded during mitosis by Mmi1 and the exosome [42], [44], [48]; sequestration of Mmi1 is a critical step in meiotic initiation, and we suggest that in *S. cerevisiae* an as-yet unidentified mitosis-specific factor directs MUT degradation by the exosome in mitosis but not meiosis.

We find that loss of TRAMP activity impedes meiotic DSB formation, showing that RNA quality control does play an important role in meiosis. A reduction in meiotic DSB formation would be expected to increase rates of chromosome mis-segregation and aneuploidy, and is consistent with the previously reported defects in *trf4*Δ meiosis [51], [52]. Such an increase was not observed when assessed by Petronczki *et al*. [65], but this likely stems from the temperature of meiosis; *trf4*Δ mutants are cold sensitive [51] and we observed DSB defects at 25° whereas Petronczki *et al*. performed meiosis experiments at 30°.

Although DSB formation is defective in *trf4*Δ mutants, this does not reflect a direct requirement for CUTs or Trf4 in the DSB formation process as there is no correlation between CUT locations and known DSB sites [66]. Neither does the defective DSB formation in *trf4*Δ cells stem from an obvious gene expression change or from any failure in meiotic entry or DNA replication. However, the DSB formation phenotype is very similar to that of previously reported *sae1* point mutants [67], which have been mapped to the CBC component Cbc2. Given that almost 40% of Cbc2 is aberrantly associated with CUTs in *trf4*Δ mutants it is very likely that the functions of Cbc2 are significantly impaired in these cells, providing a simple explanation for the observed DSB phenotype. Examination of the Cbc2-associated RNA profiles published by Neil *et al*. [53] for mitotic *trf4*Δ *rrp6*Δ cells reveals a similar predominance of CUT transcripts, showing that this is a general phenomenon. The CBC is involved in RNA export, transcription, splicing and degradation in yeast and mammals [15], [68]–[73] and it is unsurprising in this context that the exosome and CUT targeting complexes physically interact with the CBC [31], [74]. We suggest that cells defective in CUT degradation have general defects in RNA processing due to saturation of the CBC complex, which may explain many of the currently unattributed phenotypes displayed by TRAMP and exosome mutants.

## Materials and Methods

### Yeast Strains and Culture Conditions

Yeast strains listed in Table S2 were created in the SK1 background by standard methods using the oligonucleotides in Table S3 and pFA6a series plasmids. For synchronised sporulation, cells from 15% glycerol stocks were plated onto YPGly (YP+2% glycerol) plates and grown over night. Cells were transferred to YP4D (YP+4% glucose) plates and grown for 4–6 hours before inoculating 25 ml YPD cultures. Cultures were grown over night at 30°, and diluted the following afternoon into YPA (YP+2% KOAc) at 2.6×10^6^ cells/ml or 5×10^6^ cells/ml for wt and *trf4*Δ cells respectively. Cells were grown for ∼18 hours at 30° with shaking at 200 rpm, then washed and re-suspended in SPO medium (3 g/L KOAc, 5 mg/L uracil, 5 mg/L histidine, 25 mg/L leucine, 12.5 mg/L tryptophan, 200 mg/L raffinose) at 4×10^7^ cells/ml and incubated at 25°C with shaking at 250 rpm. Media components were purchased from Formedium and Sigma.

### RNA extraction and analysis

Cells were fixed by addition of ethanol to 70%, then RNA was extracted using the GTC-phenol method, northern blotting was performed as described using probes listed in Table S4 [75].

### Cbc2 immunoprecipitation and high-throughput sequencing

Cbc2 IPs were performed on 1.6×10^10^ cells using IP method 1 (see below), except that TEV elution was for 2 hours at 16° and calmodulin binding was for 50 min at 4°. To prepare RNAseq libraries, RNA was fragmented in 200 mM Tris acetate pH 8.2, 500 mM KOAc, 150 mM MgOAc_2_ for 3 min at 94°, ethanol precipitated and reverse transcribed from random hexamers using Superscript II (Life Technologies) according to manufacturer's instructions. Tris pH 7.8 and MgCl_2_ were added to 50 mM and 5 mM respectively, and second strand synthesis was performed with 2U RNase H (NEB) and 50U DNA polymerase I (NEB) at 16° for 2.5 hours. Ends were repaired using 15U T4 DNA polymerase (NEB), 5U Klenow DNA polymerase (NEB) and 50U T4 PNK (NEB) in T4 DNA ligase buffer (Life Technologies) at 20° for 30 min. cDNA was tailed with dATP using 15U Klenow (3′-5′ exo-) (NEB) in NEBuffer 2 at 37° for 30 min, and 1∶10 diluted Illumina adapters were ligated using a Ligafast kit (Promega) followed by gel purification of ∼200 bp species. Libraries were amplified for 15 cycles using Phusion polymerase (NEB) and Illumina PCR primers 1.0 and 2.0. Wild type and *trf4*Δ samples were sequenced on separate lanes of an Illumina GA-II system. Reads were mapped to the yeast reference genome (SGD1.01) or a custom assembled SK1 genome using Bowtie [76], allowing either unique mapping reads only or allowing non-unique reads to map at random respectively. For analysis, reads were summed in 100 bp segments spanning the genome using SeqMonk (http://www.bioinformatics.babraham.ac.uk/projects/seqmonk/), and reads from the 37S pre-rRNA were filtered out of the analysis as these represent an abundant contaminant of non-Cbc2-bound transcript. Total read-count normalisation was then applied to account for differing sequencing depths (16 million mapped reads for wild type, 21 million for *trf4*Δ). Analyses were performed using an R script (File S1). Sequencing data is deposited at GEO, accession number GSE60221.

### Protein Extraction and western blotting

2×10^7^ cells were washed once with water and resuspended in 100 µl water. 15 µl 2 M NaOH with 80 mM DTT was added, the suspension was mixed by vortexing and incubated for 10 minutes on ice. 15 µl 50% TCA was added and the suspension vortexed and incubated for a further 10 minutes on ice. Samples were centrifuged for 2 minutes at 10,000 *g* and the pellet was washed with acetone and dried for 10–20 minutes at room temperature. The pellet was resuspended in 20 µl sample buffer (100 mM Tris pH 6.8, 2% SDS, 0.1% Bromophenol Blue, 10% glycerol, 100 mM DTT), vortexed and boiled at 95°C for 5 minutes. Proteins were separated on 8% or 12% polyacrylamide gels and transferred to a nylon membrane (LI-COR) using the NOVEX system (Invitrogen). Antibody staining was performed using standard methods for HRP or fluorescent detection (see protocols at www.cellsignal.com) and imaged using film or a LI-COR Odyssey system. Primary antibodies: mouse anti-Pgk1 (Invitrogen 459250) 1∶10,000, rabbit anti-TAP (Open Biosystems CAB1001) 1∶200-1∶1000 depending on the batch, peroxidase anti-peroxidase (Sigma P1291) 1∶5000, rabbit anti-GFP (Abcam ab290) 1∶2000.

### Co-immunoprecipitation

IP method 1: 1.75×10^10^ log phase or 4×10^10^ sporulating cells were harvested and washed with PBS. The cells were resuspended in one pellet volume of lysis buffer (50 mM HEPES pH 7.5, 50 mM KCl, 5 mM MgCl_2_, 1 mM DTT, cOmplete Mini EDTA-free Protease Inhibitors) and frozen drop-wise on liquid nitrogen. Cells were ground to a fine powder in a pestle and mortar under liquid nitrogen and thawed in a 50 ml tube on ice. The lysate was centrifuged for 5 min at 4500 rpm and the supernatant clarified by centrifugation for 20 min at 30,000 *g*. Cleared lysate was transferred to a 15 ml tube and incubated with 250 µl/100 µl (log phase/sporulating samples respectively) of IgG sepharose beads (G.E. Healthcare) for 2 hours at 4°C with gentle agitation. Beads were washed five times for 10 minutes at 4°C with IP Buffer (10 mM Tris pH 7.5, 120 mM NaCl, 5 mM MgCl2, 0.1% NP-40, 1 mM DTT). Beads were incubated overnight at 4°C with 250 µl/100 µl IP buffer and 2.5 µl/1 µl AcTEV protease (Invitrogen). Beads were removed by centrifuging through a Micro Bio-Spin column (Bio-Rad), and the flow-through was diluted with 500 µl IP buffer +2 mM CaCl_2_ and incubated for 1.5 hours at 4°C with 100 µl calmodulin sepharose beads (G.E. Healthcare). Beads were washed three times for five minutes with IP buffer +2 mM CaCl_2_ and protein was eluted in 100 µl calmodulin elution buffer (10 mM Tris pH 7.5, 120 mM NaCl, 5 mM EGTA). Beads were removed by centrifuging through a Micro Bio-Spin Column. 50 µl protein was analysed by SDS-PAGE followed by silver staining using the SilverQuest™ Silver Staining Kit (Invitrogen) and 20 µl was analysed by electrospray Mass Spectrometry using a Thermo LTQ Orbitrap Velos.

IP method 2: 1.75×10^10^ log phase or 4×10^10^ sporulating cells were harvested in a 50 ml falcon tube and washed with PBS. The cell pellet was resuspended in 1 pellet volume of TMN150 (50 mM Tris pH 7.8, 150 mM NaCl, 5 mM MgCl_2_, 0.1% NP-40) with protease inhibitors (5 µg/ml Chymostatin, 5 µg/ml Leupeptin, 5 µg/ml Antipain, 5 µg/ml Aprotinin, 5 µg/ml Pepstatin A, 5 µg/ml E-64, 2 mM AEBSF, 1 mM Benzamidine, 1 mM PMSF, 2 mM NEM, 200 µM ALLN) and 5 mM β-Mercaptoethanol. 2–3 pellet volumes of Zirconium beads were added and the cells were lysed by 6 cycles of vortexing for 1 minute followed by 1 min on ice. The lysate was centrifuged for 20 min at 4,600 *g* then the supernatant was transferred to a 2 ml tube and centrifuged for 20 min at 20,000 *g*. The supernatant was incubated with 0.3 ml IgG sepharose for two hours at 4°C. Beads were washed four times for 5 min with TMN150 and incubated overnight in 600 µl TMN150 with 6 µl AcTEV protease (Invitrogen) and 5 mM β-Mercaptoethanol. 100 µl calmodulin sepharose beads (G.E. Healthcare) and 2 mM CaCl_2_ (final volume) were added, and the sample was incubated for 2 hours at 4°C on a rotating wheel. Beads were washed four times for 5 min with TMN150+2 mM CaCl_2_ and protein was eluted and processed as per IP protocol 1. Mass spectrometry data is deposited at the PRIDE archive, accession numbers PXD001239 and PXD001223.

### RT PCR

1 µg RNA was treated with 1 µl RQ1 DNase (Promega) for 30 minutes at 37°C then re-purified by phenol-chloroform extraction followed by ethanol precipitation. The re-purified RNA was re-suspended in 12 µl water and heated to 65°C for five minutes with 250 ng random hexamers then left to cool on ice. 4 µl 5× First Strand Buffer (Invitrogen) and 1 µl 0.1 M DTT (Invitrogen) were added and the reaction was divided equally into 9.5 µl+ and - RT samples. 0.5 µl SuperScript III (Invitrogen) was added to the +RT sample and both samples were incubated for 10 minutes at room temperature followed by 30 minutes at 50°C. Samples were heat inactivated for 15 minutes at 70°C and diluted with 40 µl water. PCR reactions were performed in 20 µl volumes using Phire polymerase, primers are listed in Table S3.

### FACS analysis

Cells were fixed in 70% ethanol overnight at −20°C. 6x10^6^ fixed cells were washed and resuspended in 500 µl FACS wash buffer (50 mM tri-sodium citrate, pH 7.0) then sonicated briefly and treated with 1 mg/ml RNase A in FACS wash buffer for 2–3 hours at 37°C. Cells were washed with FACS stain buffer (200 mM Tris pH 7.5, 211 mM NaCl, 78 mM MgCl_2_) and stained overnight at 4°C in 450 µl FACS stain buffer and 50 µl 0.5 mg/ml propidium iodide. Cells were sonicated again and diluted with 500 µl FACS wash buffer. FACS was performed using a FACSCalibur (BD) in accordance with the manufacturer's instructions. Data analysis was performed using CellQuest software.

### PFGE analysis

Cells were fixed in 70% ethanol, then processed, separated and probed as described [77].

## Supporting Information

Figure S1 **Characterisation of homozygous SK1** ***trf4*** **Δ diploids.** A: Growth of SK1 wild-type and *trf4*Δ diploids at 30°, 25° and 18° on YPD plates. B: Tetrad formation in SK1 wild-type and *trf4*Δ cells grown at 30° in YPA then shifted to sporulation media at 25° for 24 hours. C: Induction of *SPO11* during meiosis in wild-type and *trf4*Δ cells assayed by northern blot. Graph shows average of data from two independent experiments, error bars indicate ±1 s.d.
(TIF)

Figure S2 **Validation of Cbc2-TAP immunoprecipitation protocol.** A: Western blot showing purification of Cbc2-TAP from meiotic wild-type and *trf4*Δ cells. After lysis and clarification, a sample was taken for total protein (lanes 3,4) while the remaining sample was subjected to a two-step TAP purification protocol. Lanes 1,2 show lysate after binding to IgG beads, lanes 5,6 and 9,10 show material remaining on IgG and calmodulin beads after elution. Lanes 11,12 show final product. B: Northern blot of total, unbound and Cbc2-TAP associated RNA from meiotic wild-type and *trf4*Δ cells probed for *NEL025*, *ACT1* and 18S. C: Scatter plot of log-transformed read counts from Cbc2-associated RNA isolated from wild-type and *trf4*Δ cells after six hours of meiosis. Red dots indicate points from the region Chr. V:10–40 kb that is shown in Figure 2C.
(TIF)

Figure S3 **Chromosome-wide distribution of Cbc2-associated RNA.** Distributions of Cbc2-associated RNA in wild-type and *trf4*Δ cells across chromosomes I, II, III and VI, as Figure 2D.
(TIF)

Figure S4 **Activity of P_GAL_-** ***GFP-RRP6*** **construct.** A: Northern blot of RNA from spores of the P*_GPD1_*-*GAL4*-*ER* P*_GAL1_*-*GFP*-*RRP6* strain grown to mid-log in YPD or YPGal media. RNA was separated on an 8% denaturing PAGE gel before probing for 5.8S+30, a 3′ extended 5.8S processing intermediate that accumulates in *rrp6*Δ mutants. Ethidium staining of 5S and 5.8S is shown as a loading control. The strain is heterozygous for P*_GAL1_-GFP-RRP6*, and therefore two out of four spores accumulate 5.8S+30 when grown in glucose (where P*_GAL1_* is repressed) but not in galactose. B: Western blot showing that full length GFP-Rrp6 protein is produced after estradiol induction, in addition to some degradation products. Proteins were separated on an 8% gel and probed for GFP. Ponceau-stained total protein on the same membrane is shown as a loading control. FL indicates the full length protein band.
(TIF)

Table S1 **CUT transcripts identified in meiotic cells.** Meiotic transcripts over-represented in *trf4*Δ cells compared to wild-type. Cbc2-associated RNA was isolated from cells at meiosis t = 6 hours and sequenced, reads were collected in 100 bp bins and regions of three or more consecutive bins over-represented by at least 4-fold in *trf4*Δ RNA were annotated as CUTs, see materials and methods for more information. Enrichments are quoted as log_2_ of actual values.
(XLS)

Table S2 **Yeast strains used in this study.**
(XLS)

Table S3 **Oligonucleotides used in this study.**
(XLS)

Table S4 **Hybridisation probes used in this study.**
(XLS)

File S1 **R script for analysis of sequencing data.** Script used to normalise read counts and discover CUTs, executed in R v3.0.2.
(TXT)
