# Supplementary figures and images for: The Nuclear Exosome Is Active and Important during Budding Yeast Meiosis
Source: PLoS One. 2014 Sep 11;9(9):e107648. doi: 10.1371/journal.pone.0107648 (PMC4161446; doi:10.1371/journal.pone.0107648)

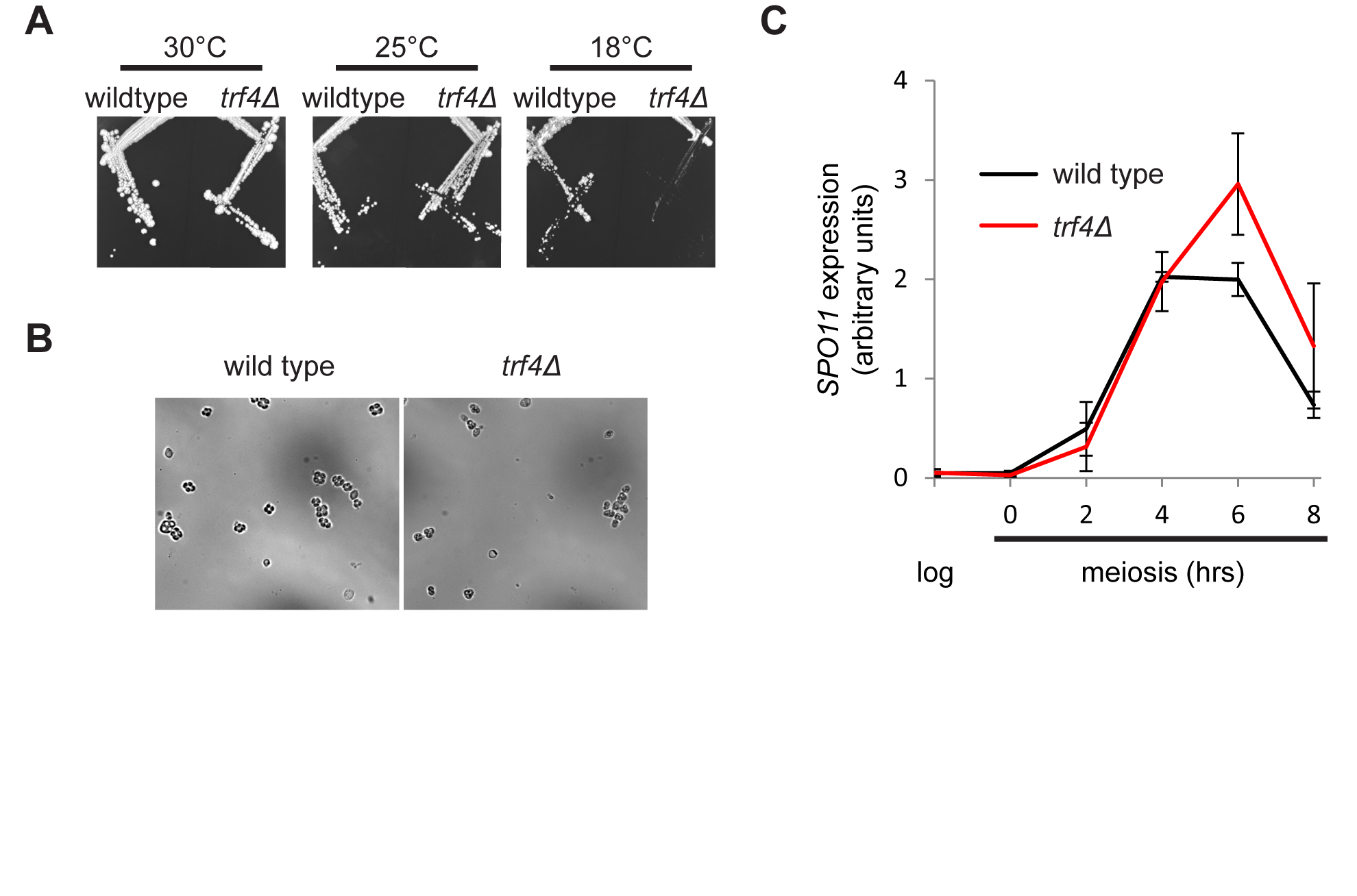

Supplement: Figure S1 — Characterisation of homozygous SK1 trf4 Δ diploids. A: Growth of SK1 wild-type and trf4Δ diploids at 30°, 25° and 18° on YPD plates. B: Tetrad formation in SK1 wild-type and trf4Δ cells grown at 30° in YPA then shifted to sporulation media at 25° for 24 hours. C: Induction of SPO11 during meiosis in wild-type and trf4Δ cells assayed by northern blot. Graph shows average of data from two independent experiments, error bars indicate ±1 s.d. (TIF) [file pone.0107648.s001.tif]

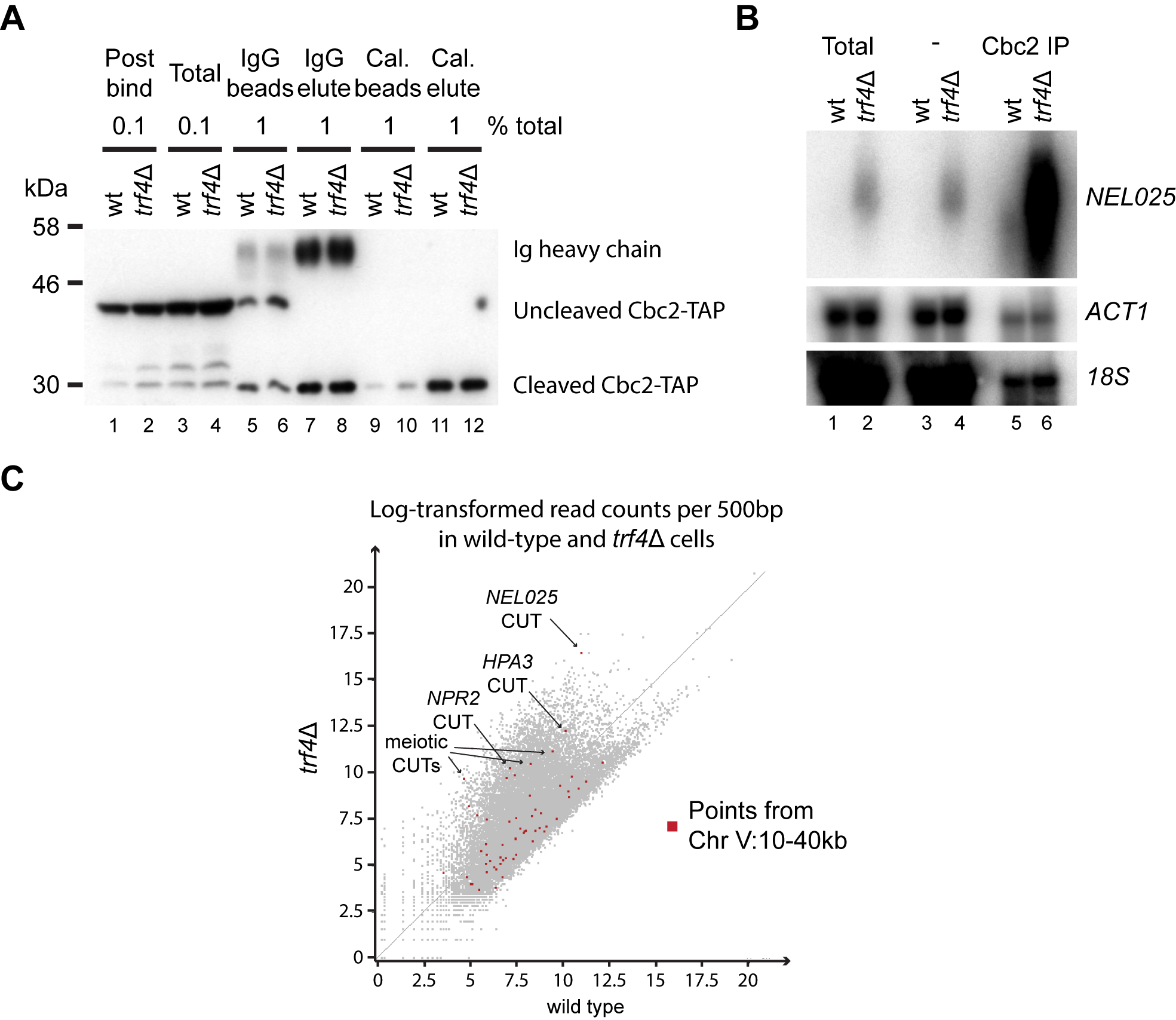

Supplement: Figure S2 — Validation of Cbc2-TAP immunoprecipitation protocol. A: Western blot showing purification of Cbc2-TAP from meiotic wild-type and trf4Δ cells. After lysis and clarification, a sample was taken for total protein (lanes 3,4) while the remaining sample was subjected to a two-step TAP purification protocol. Lanes 1,2 show lysate after binding to IgG beads, lanes 5,6 and 9,10 show material remaining on IgG and calmodulin beads after elution. Lanes 11,12 show final product. B: Northern blot of total, unbound and Cbc2-TAP associated RNA from meiotic wild-type and trf4Δ cells probed for NEL025, ACT1 and 18S. C: Scatter plot of log-transformed read counts from Cbc2-associated RNA isolated from wild-type and trf4Δ cells after six hours of meiosis. Red dots indicate points from the region Chr. V:10–40 kb that is shown in Figure 2C. (TIF) [file pone.0107648.s002.tif]

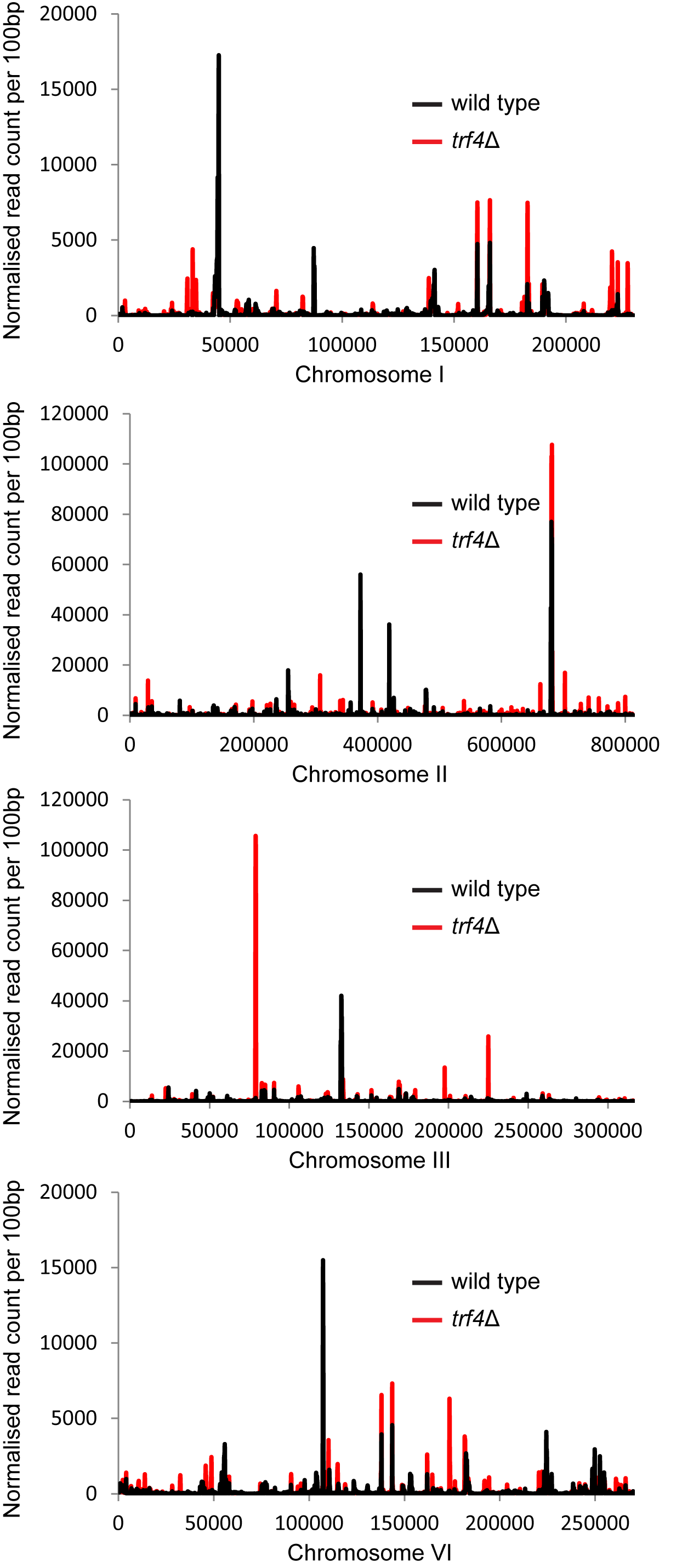

Supplement: Figure S3 — Chromosome-wide distribution of Cbc2-associated RNA. Distributions of Cbc2-associated RNA in wild-type and trf4Δ cells across chromosomes I, II, III and VI, as Figure 2D. (TIF) [file pone.0107648.s003.tif]

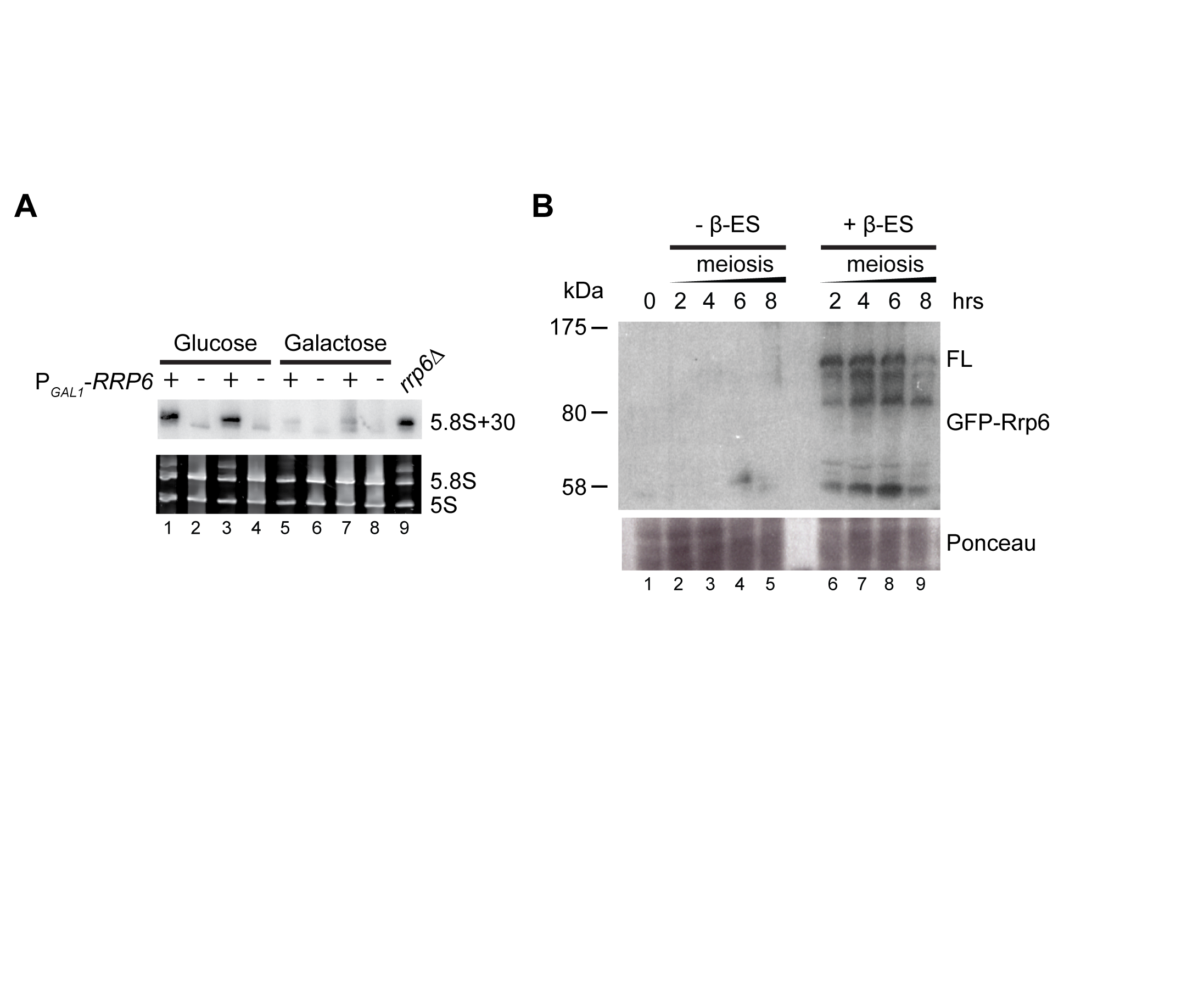

Supplement: Figure S4 — Activity of PGAL- GFP-RRP6 construct. A: Northern blot of RNA from spores of the PGPD1-GAL4-ER PGAL1-GFP-RRP6 strain grown to mid-log in YPD or YPGal media. RNA was separated on an 8% denaturing PAGE gel before probing for 5.8S+30, a 3′ extended 5.8S processing intermediate that accumulates in rrp6Δ mutants. Ethidium staining of 5S and 5.8S is shown as a loading control. The strain is heterozygous for PGAL1-GFP-RRP6, and therefore two out of four spores accumulate 5.8S+30 when grown in glucose (where PGAL1 is repressed) but not in galactose. B: Western blot showing that full length GFP-Rrp6 protein is produced after estradiol induction, in addition to some degradation products. Proteins were separated on an 8% gel and probed for GFP. Ponceau-stained total protein on the same membrane is shown as a loading control. FL indicates the full length protein band. (TIF) [file pone.0107648.s004.tif]
